# Comparison of short-term (saliva) and long-term (hair) cortisol levels in out-patients with melancholic and non-melancholic major depression

**DOI:** 10.1192/bjo.2020.8

**Published:** 2020-04-23

**Authors:** Andrés Herane-Vives, Allan H. Young, Toby Wise, Juan Aguirre, Valeria de Angel, Danilo Arnone, Andrew Papadopoulos, Anthony J. Cleare

**Affiliations:** Centre for Affective Disorders, Department of Psychological Medicine, Institute of Psychiatry, Psychology & Neuroscience, King's College London, UK; and Neuroscience and Mental Health Group, Institute of Cognitive Neuroscience, University College London, UK; Department of Psychological Medicine, Institute of Psychiatry, Psychology and Neuroscience, King's College London & South London and Maudsley NHS Foundation Trust, Kent, UK; Unidad de Trastornos Bipolares, Departamento de Psiquiatría, Facultad de Medicina, Pontificia Universidad Católica de Chile, Chile; Centre for Affective Disorders, Department of Psychological Medicine, Institute of Psychiatry, Psychology & Neuroscience, King's College London, UK; College of Medicine and Health Sciences, Department of Psychiatry, United Arab Emirates University, United Arab Emirates; and Centre for Affective Disorders, Department of Psychological Medicine, Institute of Psychiatry, Psychology & Neuroscience, King's College London, UK

**Keywords:** Depressive disorders, biomarkers, melancholic, non-melancholic, hair cortisol

## Abstract

**Background:**

Major depressive episodes (MDEs) show diverse cortisol level alterations. Heterogeneity in symptom profiles, symptom severity and cortisol specimens may explain these heterogeneous results. Less severely ill out-patients with a non-melancholic MDE (NM-MDE) may have a variation in the rhythm of cortisol secretion rather than in its concentration.

**Method:**

Cortisol measures were taken (a) over a short-term period (12 h) by measuring daily salivary output using the area under the curve with respect to the ground (AUCg) and (b) over a long-term period (3 months) in hair. Additionally, cortisol reactivity measures in saliva – the cortisol awakening response and the 30 min delta cortisol secretion after awakening (DELTA) – were investigated in 19 patients with a melancholic MDE (M-MDE) and 52 with a NM-MDE, and in 40 matched controls who were recruited from the UK and Chile. Depression severity scores were correlated with different cortisol measures.

**Results:**

The NM-MDE group showed a decreased AUCg in comparison with controls (*P* = 0.02), but normal cortisol reactivity and long-term cortisol levels. The M-MDE group did not exhibit any significant cortisol alterations nor an association with depression severity scores. Higher Hamilton Rating Scale for Depression score was linked with decreased hair cortisol concentration (HCC, *P* = 0.05) and higher DELTA (*P* = 0.04) in NM-MDEs, whereas decreased HCC was the sole alteration associated with out-patients with severe M-MDEs.

**Conclusions:**

The contrasting short- and long-term cortisol output results are compatible with an alteration in the rhythm of cortisol secretion in NM-MDEs. This alteration may consist of large and/or intense episodes of hypercortisolaemia in moderate NM-MDEs and frequent, but brief and sharp early-morning DELTAs in its severe form. These changes may reflect the effects of environmental factors or episodes of nocturnal hypercortisolaemia that were not measured by the short-term samples used in this study.

## Background

Inconsistencies remain within the literature regarding cortisol levels in patients with a major depressive episode (MDE). Increased cortisol levels have been suggested as a potential biomarker for an MDE,^1^ but not all studies have confirmed this finding.^2^ This may be, in part, explained by the considerable heterogeneity inherent in the depressive syndrome. Indeed, it has been shown that subtypes of depressive episodes with typical neurovegetative features,^3^ such as psychotic or melancholic MDE (M-MDE)^4–6^ largely explain the increased cortisol levels found in this disorder. In turn, these depressive features are mainly found in in-patients who are more severely ill.^4,5^

Many studies have shown that non-melancholic MDEs (NM-MDEs), including specific subtypes such as atypical MDEs in particular, but also more moderate-severity forms of depression and individuals without major neurovegetative alterations or somatic symptoms, are associated with low cortisol levels.^7,8^ Importantly, several studies have shown that, although bearing the name ‘atypical’, this subtype of depression is the most common clinical presentation in out-patients.^9–11^

## Measuring cortisol levels

As a result of the reactivity and diurnal variability of this hormone, it has been difficult to find a measure capable of accurately capturing long-term cortisol secretion levels. However, hair specimens provide a relatively novel way in which to measure chronic levels of cortisol^12^ and are a potential advance on current methods of assessment, since hair specimens are not affected by many of the various confounds in cortisol assessments.^13–17^ Previous studies have reported a positive correlation between hair and saliva specimens that becomes significantly stronger as the number of saliva samples increases.^18^

## Cortisol levels and MDEs

The nature of cortisol secretion may also provide an additional explanation for the lack of consistency in studies of cortisol levels in MDEs. Using a series of measures of short-term cortisol levels it has been shown that out-patients with MDEs may present with daily variations in the rhythm of cortisol secretion, rather than in its absolute concentration.^2^ In fact, we recently published data suggesting that this neurobiological feature may also describe the long-term pattern of cortisol secretion in atypical MDEs, after using a combination of hair and saliva measures.^19^ However, we do not know whether this pattern also describes other non-melancholic forms of MDEs. Although more studies have investigated hair cortisol levels in different types of depression,^20–22^ to date, no affective disorder research study has used a combination of hair and saliva measures, and compared non-melancholic versus melancholic major depression.

Taken together, this previous research emphasises the importance of taking into account the use of different specimens for measuring cortisol, variable depression severity and different subtypes of depression in order to provide further clarity with regard to cortisol status in MDEs. However, episode severity has a potential effect not just on cortisol levels but also cortisol reactivity. For example, although some authors have found that more severe symptoms were associated with increased cortisol reactivity,^23^ the same variable has been negatively associated with long-term cortisol secretion levels.^24^ Indeed, some studies that attempted to correlate hair cortisol concentration (HCC) with severity of depression scores, showed either a negative association or no association.^25,26^ However, three prior hair studies used either (a) a mixed sample of in-patients and out-patients^24,25^ or (b) patients with MDEs presenting with several additional psychiatric comorbidities or taking psychotropic medication.^24,27^ These are all potentially confounding factors when measuring cortisol levels.

## Aims

Thus, we decided to re-analyse our data as a result of the conclusions of our most recent work^19^ in order to answer these new questions and hypotheses. We designed this study with three objectives:

- To study a full set of cortisol reactivity and secretion measurements using contemporaneously obtained short- and long-term measures in out-patients with depression who were not taking medication and who had no additional psychiatric comorbidities. We compared cortisol levels from these patients with an age- and gender-matched group of healthy controls.
- To compare cortisol levels using the same cortisol measures in individuals with an M-MDE and those with an NM-MDE.
- To investigate whether the same measures of cortisol are influenced by differences in depression severity scores.

We hypothesised that out-patients with MDEs with non-melancholic features would show an abnormal long-term rhythm of cortisol secretion in comparison with healthy controls. More severe forms of NM-MDE would be associated with increased short-term reactivity but decreased long-term cortisol secretion levels.

## Method

### Participants

Participants were recruited in London (UK) and Santiago (Chile) from public advertisements^28^ and local psychological therapy services. All patients were assessed by researchers (A.H.-V, D.A and T.W. in the UK; V.d.A. in Chile) who had undergone common training in the methodology and research tools. All participants were assessed with psychometric scales validated in both English and Spanish. Diagnostic assessment was completed with the Mini International Neuropsychiatric Interview (MINI).^29^ Patients were required to meet DSM-IV^30^ criteria for an MDE in the context of either a unipolar or bipolar disorder. As DSM-5^31^ was released during the study, we updated to use DSM-5 criteria and confirmed that all previously enrolled participants would also have met the DSM-5 criteria. In total, 71 patients with an MDE were recruited from the UK (*n* = 45) and Chile (*n* = 26).

The subclassification of patients was based on DSM-5 subtype specifiers. Thus, patients who met criteria for either melancholic or psychotic depression were considered to have an M-MDE,^32^ whereas all others were considered an NM-MDE. Using these criteria, the sample comprised 52 participants in the NM-MDE group (38 women, mean age  31.8 years and mean body mass index (BMI) 26.4 kg/cm^2^) and 19 participants in the M-MDE group (10 women, mean age 38.2 years and mean BMI  24.3 kg/cm^2^). Melancholic symptoms were also measured using the Newcastle Depression Diagnostic scale.^33^ The presence of symptoms of elation and other hypomanic features were excluded using the Young Mania Rating Scale (YMRS).^34^

The control group comprised 40 individuals (29 women, mean age 33.2 years and mean BMI  24.3 kg/cm^2^) who were recruited from public advertisements and from hospital and university staff across the two sites: UK (*n* = 32) and Chile (*n* = 8). Healthy controls were assessed to ensure they had no current or past psychiatric diagnoses and reported no history of psychiatric illness in first-degree relatives. Controls were further selected in order to match on demographic variables and potential biological confounders (BMI, waist circumference, frequency of hair washing and use of contraceptives, tobacco, alcohol and cosmetic treatments) as closely as possible with the participants with depression.

Depression severity was established using the Hamilton Rating Scale for depression – 17 item version (HRSD-17).^35^ A severity criterion on the HRSD-17 was not imposed given our hypotheses in relation to depression severity as a continuous moderating variable. We did, however, require patients to have a score ≥11 to ensure they had clinically significant ongoing depressive symptoms. Depressive symptom ratings using this scale were evaluated on an independent set of patients and showed high interrater reliability across sites (intraclass correlation coefficient 0.96, *P* < 0.01).

For all participants, the frequency and severity of the most common day-to-day environmental disturbances during the month prior to study enrolment were measured using the Hassles Scale.^36^ More unexpected environmental factors, such as major life events during the 3 months prior to the study, were assessed using the Recent Life Changes Questionnaire (RLCQ).^37^ The occurrence of early life stressors (trauma) was assessed using the Childhood Trauma Questionnaire (CTQ).^38^ The presence of childhood trauma was recorded if a participant presented with a score greater than the threshold on any of the following subscales of the CTQ: emotional abuse (threshold >12), physical abuse (threshold >9), sexual abuse (threshold >7), emotional neglect (threshold >14) and physical neglect (threshold >9).

Participants had to be free of taking psychotropic medication or other medication liable to affect the hypothalamic–pituitary–adrenal (HPA) axis for at least 3 months and from ongoing psychological interventions at the time of inclusion. All participants provided a hair sample of at least 3 cm length, and the 3 cm closest to the scalp was used in the hair cortisol assay. Participants were excluded if they reported any illicit substance use in the previous 3 months or had any unstable medical condition

The research was approved by the local ethics committees in both Chile (Universidad de Chile) and in the UK (King's College London) and written informed consent was obtained from all participants. All participants were compensated for taking part in the research.

### Materials

#### Hair specimens

A trained practitioner collected hair samples of all participants. Hair samples were collected from the vertex using standardised procedures, including recording the presence and frequency of confounders potentially affecting HCC, such as dyeing, bleaching, permanent straightening or waving and frequency of hair washing. The scalp end was clearly marked, and the 3 cm of hair nearest the scalp was measured and separated in the laboratory to be used for analysis. Collection procedures and analyses for each participant were standardised according to a strict protocol to produce approximately 3 months of hair growth, equivalent to a 3-month retrospective assessment of endogenous cortisol production. Cortisol levels were determined using a commercially available competitive enzyme-linked immunosorbent assay (Salimetrics LLC, USA) and the results expressed in picograms of cortisol per milligram of hair (pg/mg). All hair samples were analysed at Salimetrics Laboratory, Cambridge, UK (www.salimetrics.com)^39^ (see supplementary material available at https://doi.org/10.1192/bjo.2020.8 for procedural details).

#### Saliva specimens

Saliva samples were collected at the baseline assessment on a weekday (Tuesday to Friday) following hair sampling. Participants were asked to provide six saliva samples on a typical day using plain salivettes (Sarstedt, Leicester, UK) as per Roberts' protocol;^40^ full written instructions were given at the time of the assessment. Analyses of saliva cortisol concentrations were carried out in the Affective Disorders Laboratory at the Bethlem Royal Hospital, London UK. The area under the curve with respect to the ground (AUCg) was used for calculating the daily cortisol output using the six samples. Two measures of cortisol reactivity in saliva were analysed in this study, namely the cortisol awakening response (CAR) and the early morning delta cortisol value (DELTA). The CAR was calculated using the AUC with respect to increase (AUCi) on the first three morning saliva samples collected over a 1 h period. We also used the values provided by the 30 min post-awakening measure as an index of the highest DELTA of cortisol during the day, as this is normally the time of day with the highest cortisol levels (see supplementary material for procedural details). In addition, these two cortisol reactivity measures are less influenced by several modulatory factors^17^ and allowed us to compare our results with the recent study by Pochigaeva et al,^24^ which also compared HCC with a single short-term cortisol measure.

### Statistical analysis

All data were analysed using Stata Statistical Software version 6.0. Demographics, clinical features and questionnaire measurements were compared using a *t-*test for continuous variables and chi*-*squared or Fisher's exact test for categorical variables. Cortisol data were checked for normality using graphic methods, including histograms and Shapiro–Wilk test.

The use of this statistical test revealed that all salivary cortisol levels were normally distributed across the control group, the entire patient group with MDE (MDE-total group), those patients with M-MDEs (M-MDE group) and those with NM-MDEs (NM-MDE group) (all *P* > 0.05). However, *P*-values were <0.01 for HCC in the control, MDE-total and NM-MDE groups and *P* = 0.05 for the M-MDE group. Furthermore, as the power of statistical tests for testing normality are usually low when the sample size is small,^41^ as in the M-MDE group here, we mostly report non-parametric results (Mann–Whitney *U*-test). Notwithstanding, we did not find divergent results between these two types of analyses provided (see supplementary Results). However, we report the results provided by parametric (ANOVA) and non-parametric (Kruskal–Wallis) tests, when cortisol results were compared between the three groups: control, NM-MDE and M-MDE, since only ANOVA test allows *post hoc* corrections using the Bonferroni test for multiple comparisons.

ANCOVA was used to control for any biological confounder potentially affecting cortisol level measurements among groups. A linear regression model was used to evaluate the relationship between depression severity scores on the HRSD-17 and salivary cortisol levels in the MDE-total, M-MDE and NM-MDE groups. We used generalised linear models with a gamma distribution and a log-link function to model the relationship between HRSD-17 scores and HCC in the MDE-total, M-MDE and NM-MDE groups. The level of significance was set at *P* < 0.05 (two-tailed) for all tests.

## Results

Detailed demographic and clinical variables of the participants are presented in supplementary Table 1. As was predicted, most of our participants with depression had a moderate episode severity^42^ with non-typical features and a unipolar longitudinal history (all *P* < 0.01). None of the patients showed significant current hypomanic symptoms, using the YMRS scale. They had been exposed to high levels of environmental factors during either their early life in the form of childhood trauma (CTQ), or more recently, as major life events (RLCQ) or minor daily hassles (The Hassles Scale). The UK site provided the majority of patients (45 (63.4%) and controls (32 (80.0%)) (supplementary Table 1).

There was no difference in BMI between the control and the MDE-total group, but women in the control group were significantly more likely to be taking contraceptive pills than women in the MDE-total group (*P* = 0.03). Confounding factors for hair and saliva cortisol levels did not differ among the M-MDE, NM-MDE and control groups, apart from age as participants in the M-MDE group were older overall than those in the NM-MDE group (see additional clinical features in supplementary Table 1)

In relation to the differences in severity scores, only controls differed in their HRSD-17 scores compared with the M-MDE and NM-MDE groups, whereas these two groups did not differ significantly from one another, both having mean scores within the moderate range of an MDE. HRSD-17 severity scores were: mean 0.3, s.d. = 0.9 for the control group; mean 17.6, s.d. = 6.5 for the M-MDE group; and mean 17.2, s.d. = 4.8 for the NM-MDE group; (*F* (2108) = 199.39, *P* < 0.01) using the ANOVA test.

### HCC in the MDE-total, M-MDE and NM-MDE groups

Analyses of HCC in the MDE-total group (*n* = 71) (mean 8.3 pg/mg, s.d.= 4.6) versus healthy individuals (mean 8.3 pg/mg, s.d. = 3.9) did not show a significant group difference, *P* = 0.96. HCC did not differ between M-MDE and NM-MDE (supplementary Table 2). Correlation analysis between the use of contraceptives and HCC did not show a significant association (*r* = 0.05, *P* = 0.66).

### Saliva cortisol concentrations in MDE-total, M-MDE and NM-MDE groups

There was a trend towards a significant reduction in the total daily salivary cortisol output (AUCg) in the MDE-total group (mean 105.8 nmol/l⋅h, s.d. = 34.8 ) versus healthy controls (mean  120.8 nmol/l⋅h, s.d. = 38.9); *P* = 0.09 (supplementary Table 2). Analysis of covariance (ANCOVA) showed that contraception did not affect AUCg values among groups (*P* = 0.08). Both CAR and DELTA did not reveal significant differences in any comparison (all *P* > 0.05) (supplementary Table 2).

Both an ANOVA (*F*(2,82) = 4.15, *P* = 0.02) (supplementary Fig. 1) and a Kruskal–Wallis tests (*P* = 0.01) showed a group effect in AUCg. A Bonferroni *post hoc* correction indicated that NM-MDE had a significantly lower AUCg in comparison with the control group (supplementary Fig. 1). This effect remained significant when age was included as a covariate (*F*(3,81) = 4.17, *P* < 0.05). Again, neither CAR nor DELTA levels showed significant differences between the two subtypes of MDE (all *P* > 0.05 using either ANOVA or Kruskal–Wallis tests) (supplementary Table 2).

### Associations between depression severity scores and cortisol levels

Associations between depression severity scores, using the HRSD-17 scale, and different cortisol measures showed dissimilar results in the MDE-total group. Whereas increases in HRSD-17 scores exhibited a significant and positive association with higher DELTA (*P* = 0.02), only a positive trend was observed between AUCg levels in short-term saliva samples and the HRSD-17 (*P* = 0.07). However, a contrasting pattern was seen using long-term hair samples, since a higher HRSD-17 score was significantly correlated with lower HCC (*P* = 0.01). On the other hand, no associations were detected between CAR and HRSD-17 (*P* > 0.05) (supplementary Figs 2 and 3).

Different results were found again when these cortisol measures were analysed separately in the NM-MDE and M-MDE groups using the same depressive symptom scores. Indeed, whereas a significant association for decreasing long-term cortisol levels using hair samples in the M-MDE group (*P* = 0.04) (supplementary Fig. 2) was found, no other significant association was found using either short-term cortisol output (AUCg) (supplementary Fig. 2) or reactivity (DELTA and CAR) levels (supplementary Fig. 3) in the same group (both *P* > 0.05). However, not only did participants in the NM-MDE group exhibit a negative and significant association between their HRSD-17 scores and their HCC levels (*P* = 0.05) (supplementary Fig. 2), but also the same scores were significantly linked to decreased early morning DELTA of cortisol (*P* = 0.04) (supplementary Fig. 3). AUCg and CAR were not associated with HRSD-17 score variations in either of the depressive subtypes that were analysed in this study (both *P* > 0.05) (supplementary Figs 2 and 3).

## Discussion

In this study we compared different measures of both cortisol reactivity and secretion in out-patients with depression who were not taking medication, with classical melancholic and non-melancholic forms of MDE, utilising both hair and saliva specimens. In comparison with controls, we found decreased short-term cortisol levels in the NM-MDE group and no difference in the M-MDE group using the same cortisol output measure. No significant difference was found using long-term cortisol secretion (HCC) or cortisol reactivity (CAR and DELTA) levels either in the whole MDE group (MDE-total) or in the subtypes studied here (M-MDE and NM-MDE).

We also analysed the effect of depression severity score on these cortisol measures. Whereas long-term cortisol secretion (HCC) showed a significant and negative association with depression severity in MDE and both depressive subtypes studied here, the short-term cortisol secretion measure used (AUCg) did not reveal any significant association with any group. One cortisol reactivity measure, the 30 min delta of cortisol after awakening, exhibited a positive and significant association with HRSD-17 score, both again in the whole depressive group and in the NM-MDE group. The other reactivity measure, the CAR measured over 60 min using the AUCi, was not related to depression severity score in any group.

### Abnormal long-term rhythm of cortisol secretion

The MDE-total group did not show differences in cortisol secretion levels in comparison with the control group, contradicting the notion that increased cortisol levels are a consistent neurobiological marker in depression as a whole.^43^ In fact, the opposite was suggested in the largest subtype studied here, in that the NM-MDE group had decreased AUCg in comparison with the control group.

However, the reduced daily cortisol output in saliva was measured over only approximately 12 h, and thus these differences in AUCg do not indicate whether low cortisol levels may be considered a chronic phenomenon in individuals with depression. In fact, the HCC findings suggest that over the long term (3 months), cortisol levels are not altered in either MDE subtype. The presence of decreased AUCg together with normal HCC in the NM-MDE group suggests that there may be episodes of increased cortisol levels on some days or some time points not measured in this study, thereby creating cumulatively normal cortisol levels later detectable in hair. However, those potential episodes may also be sporadic, as otherwise the patients with NM-MDEs would have exhibited increased rather than decreased daily cortisol output (AUCg). It may be that environmental factors trigger sporadic episodes of increased cortisol output, since we found that no other confounding factor had a significant effect on short-term cortisol levels in the NM-MDE group.

A previous study has already shown a heightened response to the Corticotrophin Releasing Hormone (CRH) test in people with NM-MDEs, a response that may mimic the effect of environmental factors.^44^ Moreover, we recently found that some environmental factors, specifically daily hassles, were indeed more commonly associated with one type of NM-MDE, atypical depression, in comparison with non-atypical subtypes.^19^ In the present study, environmental factors were also strongly associated with NM-MDEs (supplementary Table 1). Thus, it is possible that an increased reactivity to daily hassles might trigger sporadic episodes of increased cortisol output in NM-MDEs.

Alternatively, it is also possible that there are periods of increased cortisol release occurring during the night, which are undetected by the daytime salivary cortisol measures we took. Such periods may play a compensatory role for the lack of secreted cortisol during the day. However, it is also possible that nocturnal hypercortisolaemia could be related to certain depressive symptoms. Indeed, it has been shown that patients with insomnia have increased cortisol levels during the evening and first half of the night.^45^ Furthermore, some authors have also found increased nocturnal cortisol levels in participants with depression, although this was in participants with melancholic features.^46^ Either way, during the day or during the night, episodes of cortisol hypersecretion would need to be large enough or frequent enough to counter the short-term hyposecretion we found in our short-term measure in order to lead to the accumulation of normal, rather than decreased cortisol levels in hair.

Taken together, the unexpected and contrasting results found using HCC and AUCg may imply an intermittently hyperactive pattern of cortisol secretion in NM-MDEs. This may also mean that the main neuroendocrine feature for the largest subtype of depressive disorder, according to Zisook et al,^11^ Jarrett et al,^9^ and Parker et al,^10^ is an abnormal cortisol rhythm, rather than an absolute concentration alteration, as Peeters et al,^2^ had also previously claimed using series of short-term specimens. In fact, in one of our most recent studies we also suggested that this neurobiological alteration could describe the long-term pattern of cortisol secretion in atypical depression,^19^ since that depressive subtype showed the same results exhibited by the NM-MDE group here.

### CAR

Although an increased CAR could theoretically have also contributed to the accumulation of normal HCC in the NM-MDE group, in the face of the low measured daily output, we did not find a difference in the measured CAR and DELTA values in this study, either between all patients and controls, or between the NM-MDE and M-MDE groups. On a whole, our results suggest that CAR and DELTA should not be used as illness biomarkers for this moderate depressive subtype. In contrast to our findings, some previous studies have indeed found an increased CAR in patients with depression.^47,48^ The differences in the number of saliva sampling points used to calculate the CAR could explain differing results. Whereas we used three sampling points (0, 30 and 60 min), Bhagwagar et al^47^ used five (at 0, 15, 30, 45 and 60 min), after awakening, although cortisol levels were only higher at 15, 30 and 45 min post-awakening.

Vreeburg *et al*^48^ collected four saliva samples (at 0, 30, 45 and 60 min) post-awakening and again did not find cortisol variations at 0 min in comparison with controls. Thus, the normal CAR in our NM-MDE group may be explained by the few morning samples that we took in comparison with the Bhagwagar et al^47^ and Vreeburg et al^48^ studies, including, also the 0 min sample, which was the measure that both previous investigations did not find any significant variation in. It may also be possible to argue that if we had included an increased number of saliva morning samples around the 30 min delta we would had found increased, rather than normal CAR in our patients, which could, in theory, have also contributed to the accumulation of normal HCC in NM-MDEs. If that hypothesis was true, it would mean that out-patients with NM-MDEs present an abnormal endogenous cortisol reactivity featuring a shorter and delayed response.

In contrast to the above findings, Stetler & Miller^49^ found a decreased rather than increased CAR in patients with mild and moderate MDEs in comparison with controls, using salivary sampling points that were the same in number and time as our study. It is notable that the Chilean patients in our study had significantly lower AUCg in comparison with their UK counterparts and they also had significantly lower HRSD-17 scores in relation to the UK participants with depression, without exhibiting a significant difference in the presence of melancholic symptoms (see supplementary Tables 1 and 2). Different site results confirm our NM-MDE and M-MDE group results.

Finally, it seems more likely that cortisol kinetic alterations related to changes in the secretion, metabolism and/or distribution of cortisol, rather than changes in its glucocorticoid receptor activity, could explain a potential long-term cortisol rhythm in MDEs.^19^ This is because a potential glucocorticoid receptor resistance in depression would lead to enduring effects,^50^ rather than transient kinetic alterations such as the pattern of acute variations that we are speculating may be occurring.^51^ Overall, our results may focus attention on the dysregulation hypothesis of depression,^52^ which suggests that depression may be better understood as a reflection of a relative failure in its regulation, rather than as simple increases or decreases in its neurobiological compounds.

### Cortisol in out-patients with melancholic depression

Several factors may explain why we did not find increased cortisol levels in the M-MDE group on any of the measures studied here, in contrast to previous work suggesting that the melancholic and psychotic subtypes are commonly associated with hypercortisolaemia.^4–6,53,54^ First, previous studies were conducted in participants with more severe MDEs. It is clearly likely that increased cortisol levels are not a frequent finding with a less severe clinical presentation. We believe that the latter is an important point, since patients with an M-MDE in our study had, on average, depression of moderate severity; thus, our M-MDE group represented a less severe subgroup of patients with an MDE with classical features. This suggests that the severity of the episode, rather than its subtype alone, may be more critical in explaining cortisol level variations within depression, at least in out-patient settings. We believe that the aforementioned point is important, and that results might be different if we had studied a group of in-patients, who have not only more severe depression, but also more commonly present with melancholic symptoms.

Second, the use of hair specimens, as a measure of chronic cortisol concentrations is a relatively new tool and has only been used in a few studies to date. More studies are needed in order to corroborate our findings, as well as the exploration of other biological sources with the potential to measure cortisol levels over a longer-term period, such as fingernails.^55,56^ Indeed, by using fingernail specimens, we recently found increased fingernail cortisol concentration in out-patients with moderate MDEs in comparison with controls. This difference was, indeed, mainly driven by melancholic symptoms.^57^ These contrasting results could challenge the validity of hair or fingernails as long-term measures of aggregate cortisol release, or suggest that these specimens accumulate cortisol differently. Finally, as was mentioned earlier, the M-MDE group was smaller than the NM-MDE group; consequently, the power of any comparison with the M-MDE group was also reduced.

### Different effects of severity of depressive episode on cortisol levels

Of the two cortisol reactivity measures, only the morning DELTA cortisol showed a significant association with depressive symptom severity, in that increased early morning deltas of cortisol were linked with more severe episodes (supplementary Fig. 3). That association may better describe non-melancholic, rather than the classic form of depression, since the correlation was significant only in the former subtype (supplementary Fig. 3). Our findings contrast with those of Pochigaeva et al^24^ who did not find any association between the HRSD-17 score and a single morning blood cortisol measure, although they took the sample after an average of 60–80 min post-awakening, by which time physiological cortisol reactivity has already passed. The link between severity and DELTA in the NM-MDE group suggest that those early morning deltas of cortisol may be endogenous, rather than reactive to exogenous factors, and imply that the shape of the CAR is also different in more severe NM-MDE.

The positive association between DELTA and HRSD-17 score imply that the CAR in the NM-MDE group may be not only shorter and delayed, but also more pointed in more severe depression. Indeed, whereas the two previous studies^47,48^ that found increased CAR in comparison with controls used more saliva samples in their studies in comparison with us, they, at the same time, did not find an association between depression severity scores and CAR. It is quite likely, then, that an increased number of saliva samples better describe the CAR in moderate NM-MDEs, but, with severe non-melancholic depressive episodes the CAR shape becomes more pointed, mainly explained by an increase level of cortisol 30 min after awakening in comparison with the flat level at 0 min. Thus, conversely to moderate NM-MDE, it does not appear that an increased number of post-awakening saliva samples would provide further explanation of the CAR shape alteration in severe NM-MDE.

Furthermore, Adam et al,^58^ also suggested there is a predictive role of the delta, using a measure of cortisol reactivity similar to our DELTA: wake-up plus 40 min minus wake-up cortisol level. They found that higher baseline DELTA was associated with a significantly increased risk of developing depression at follow-up. Nonetheless, Pruessner et al,^59^ found that a higher CAR was associated with more severe depressive symptomatology, although this was using self-reported depressive symptoms in a sample of healthy people; accordingly, this association was only demonstrated in the non-clinical range of depression.

The association between severe NM-MDEs and DELTA suggest that those episodes may be sharp and brief, such as spikes, rather than large and/or intense events, as we suggested for its moderate form. Indeed, the integrated measure of cortisol reactivity (CAR) was not associated with severe NM-MDEs, unlike the integrated measure of cortisol secretion (AUCg) which was associated with moderate NM-MDE. This could be explained by a hyperreactive 30 min level exhausting the physiological reactivity of the HPA axis, leading to a hyporeactive 60 min level, as Bhagwagar et al^47^ found, or by a hypoactive axis at 0 min post-awakening followed by an overreacted 30 min level, as Vreeburg et al^48^ found.

Not only DELTA, but also HCC showed significant associations with HRSD-17 scores among patients with severe NM-MDE. However, HCC was linked to reduced depression severity scores. Recently, Pochigaeva et al,^24^ supporting our hair results, indicated that participants with more severe MDEs were associated with decreased long-term cortisol levels. It may be possible that those brief and sharp spikes of cortisol reactivity might also explain why severe NM-MDEs were associated with decreased, rather than normal HCC. In effect, and contrary, again to its moderate form in which the early morning DELTA of cortisol did not differ from controls, in severe NM-MDEs, those potential sharp spikes may occur often enough in order to explain why DELTA was positively associated with HRSD-17 scores. But, at the same time, they do not seem to release enough cortisol to accumulate a normal concentration in hair. By contrast, not only might they exhaust the physiological reactivity response, but they may also exhaust the adrenal gland's ability to secrete enough cortisol during the rest of the day. However, the significant association between decreased HCC and severe M-MDEs is unlikely to be explained by the potential adrenal exhaustion caused by spikes of cortisol reactivity, as there was no corresponding association between DELTA and HRSD-17 in M-MDE. Again, we do not believe that this result is likely to be clinically significant, as the severity of depression in the smaller M-MDE group does not seem to represent the classic form of the disorder.

### Limitations

It may be seen as unusual to find a group of patients with M-MDEs who have a HRSD-17 of 17.6. It has been said that melancholic features are less likely to occur in milder than in severe MDEs.^60^ However, the DSM does not include depression severity as a diagnostic criterion for an M-MDE. It highlights in its latest version that the severity of depressed mood cannot be considered as a distinct quality for melancholic mood (DSM-5). Furthermore, the author of the Newcastle scale said in 1965 that ‘the distinction between endogenous (melancholic) and neurotic (non-melancholic) forms of depression is a qualitative rather than a quantitative one’. Finally, it has been demonstrated that HRSD fails to distinguish melancholia.^61^ Nevertheless, at the same time, in-patients who are melancholic have significantly higher HRSD scores than out-patients who are melancholic.^62^ We were able to confirm that there was a variance of melancholic symptoms in our sample, after finding higher levels of melancholic symptoms as defined by the Newcastle scale within our melancholic depression group, and an extremely significant positive correlation between HRSD-17 and Newcastle scale scores (*r* = 0.65, *P* < 0.001). Having said that, the frequently observed association between melancholic symptoms and depression severity may in part occur because depression severity scales, such HRSD, overrate melancholic symptoms, whereas other depressive features, such as the reversed neurovegetative symptoms (hypersomnia and increased appetite) are mainly ignored. It is also important to note that there is a lack of data supporting the unequivocal validity of the DSM-5 specifier for melancholia, and suggestions of other criteria that may be discerning of this group of patients.^63^ Thus, we must acknowledge that the results might be different if the Newcastle scale criteria or other methods were used rather than the DSM-5 specifier, and if a group of patients with more severe depression were studied, such as in-patients, who may present not only with more severe episodes, but with more melancholic symptoms.

The effect of age was significant when AUCg values were compared between the different subtypes of MDE studied here, using an ANCOVA test. The M-MDE group was significantly older than the NM-MDE group and we found that AUCg levels were lower in the NM-MDE group in comparison with the control group. Thus, the modulatory effect that age had on short-term cortisol levels did not affect our finding of decreased AUCg levels in NM-MDEs.

Linear regression models showed that severity in the whole MDE group was associated with decreased HCC and a tendency towards increased AUCg (supplementary Fig. 2). This might seem biologically difficult to explain, since both measures were designed to assess the same cortisol profile: the total cortisol output, albeit over different periods. In effect, if those results had reached statistical significance, this would mean that hair may not be very accurate for reflecting long-term systemic levels of cortisol. In fact, it could mean, for instance, that some short-term extraneous acute influences do affect cortisol levels when hair specimens are used, whereas it has been claimed elsewhere that one of main properties of hair when accumulating substances is its immunity to those influences.^13–17^ It is true that those potentially controversial results could theoretically be explained if an MDE presented with mild hypercortisolaemia during the day and an extreme hypocortisolaemia during the night, which ended up averaging mild hypocortisolaemic levels in hair. However, we should be wary of speculating too far as, ultimately, inspecting the histograms suggests that the observed differences in AUCg and HCC in severe MDEs are potentially driven by some outliers in our control group with high levels of cortisol.

### Future directions

Taken together, our results suggest that severity, rather than different subtypes of MDEs should be used as the basis for MDE classification in out-patients. Future studies might investigate, short- and long-term cortisol levels in mild/moderate and severe MDEs in in-patients. Such a study should be conducted in particular in melancholic forms of MDEs, as our results, contrary to the large body of previous evidence,^4–6^ did not find increased cortisol secretion and/or reactivity in M-MDEs. Our study also suggests that a larger number of saliva samples should be taken in order to measure CAR in moderate NM-MDEs. Measuring its delta also seems to provide useful information about different severities of non-melancholic subtypes of depression.

Criteria for non-melancholic depression are less well defined than melancholic clinical features, and indeed, there is no operationalised definition of ‘non-melancholic’ depression *per se*. The non-melancholic group will be somewhat more heterogeneous and comprise patients with a number of subtypes of depression, including atypical, anxious-distress, peripartum onset and seasonal pattern as well as those with non-specific features. Therefore, the presence of several subtypes of major depression likely contributed to greater heterogeneity in the composition of this non-melancholic group. Future studies should use hair and saliva specimens to investigate cortisol levels in depressive subtypes. As we recently undertook such a study in atypical MDEs,^19^ the next step would be to conduct the same study in people with anxious-distressed MDEs. Such a study may reveal the effect of anxiety on cortisol levels in MDEs. Future studies might also investigate whether an abnormal long-term pattern of cortisol secretion is also a specific finding in unipolar or bipolar depression.

It is important to state that the notion of an abnormal long-term rhythm of cortisol secretion is only a hypothetical explanation, as we did not observe any of these potential episodes of increased cortisol release. Yet, based on the specimens that we have for measuring cortisol levels, the recording of a pattern of cortisol secretion in real time seems impossible to achieve. What appears to be more achievable in the near future, however, is the use of multiple specimens that accumulate cortisol over different time period in this disorder. For example, different lengths of hair or fingernail samples may be useful, particularly bearing in mind that recent studies suggest that fingernails may mainly accumulate cortisol levels when the level of this hormone is particularly high, such as during episodes of increased reactivity. This potential property of fingernails needs to be corroborated by associating, for instance, fingernail cortisol levels with the current validated specimens for measuring cortisol reactivity (CAR) and secretion (HCC and AUCg) levels. A longitudinal study design could also contribute by investigating the long-term pattern of cortisol secretion in MDEs.

### Implications

In conclusion, our results suggest that severity, rather than different subtypes of MDEs should be used as the basis for MDE classification in out-patient settings. They also strengthen the thesis that the main neurobiological finding for non-melancholic forms of depression is a cortisol rhythm, rather than an absolute concentration, alteration. This alteration may be featured by sporadic large and/or intense episodes of hypercortisolaemia in moderate NM-MDEs and frequent, but brief and sharp early morning deltas in its severe form.

## Data Availability

The datasets generated and/or analysed during the current study are not publicly available due to ongoing projects but are available from the corresponding author on reasonable request.

## Supplementary material

For supplementary material accompanying this paper visit https://doi.org/10.1192/bjo.2020.8. click here to view supplementary material
