# Supplementary material for: Comparison of short-term (saliva) and long-term (hair) cortisol levels in out-patients with melancholic and non-melancholic major depression
Source: BJPsych Open. 2020 Apr 23;6(3):e41. doi: 10.1192/bjo.2020.8 (PMC7189571; doi:10.1192/bjo.2020.8)
Supplement: Supplementary file 1 [file S2056472420000083sup001.docx]

**Supplementary Information**

**Supplementary Statistical Analysis:**

The Shapiro Wilk test revealed that, apart from DELTA (p=0.06) all UK-MDE salivary cortisol levels were abnormally distributed (all p<0.05). By contrast, and including DELTA values, all Chile-MDE salivary cortisol levels were normally distributed (all p>0.05). Therefore, the t-test was only used for comparing the deltas of MDE site’s samples, all other cortisol levels were compared using the Wilcoxon rank-sum test.

**Supplementary Results:**

The site’s proportion of M-MDE [UK: n;(%)=14;(31.1) v/s Chile: n;(%)=5(19.2)] and NM-MDE patients [UK: n;(%)=31;(68.9) v/s Chile: n;(%)=21;(80.8)] was similar (p=0.28), as well as the presence of melancholic symptoms (p=0.64) [UK-MDE Newcastle scores: Mean;(S.D)=3.5;(2.4) v/s Chile-MDE Newcastle scores: Mean;(S.D)=3.8;(2.7)]. There was, however, a significant variation in depression severity scores between sites. Although UK patients had moderate MDE [HAMD-17:Mean(S.D)=19.1;(4.9)], their scores were, on average, significantly higher than the Chilean depressed sample [HAMD-17:Mean;(S.D)=14.2;(4.6)]. No long-term cortisol alterations were found in M-MDE and NM-MDE [F (2,108) =0.20, p=0.86]. But, Chile-MDE not only exhibited decreased AUCg in comparison to controls, but also in comparison to UK-MDE (all p<0.01). HCC, DELTA and CAR did not differ between UK-MDE and Chile-MDE (All p>0.05) (Supplementary Table 2).

**Supplementary Tables & Figures:**

**Supplementary Tables:**

**Supplementary Table 1**: Demographic and Clinical Variables & Cortisol Covariates per Group.

| **Variables** | **Groups** | | *p-value* |  | |  | |  |
| --- | --- | --- | --- | --- | --- | --- | --- | --- |
|  | **Healthy Controls** | **MDE** |  | **MDE Groups** | | | *p-value* |  |
|  |  |  |  | **M-MDE** | **NM-MDE** | |  | *Post-hoc* test^ψ^ |
| **N;**  (%) | 40;  (36) | 71;  (64) | <0.01* | 19;  (17.1) | 52;  (46.9) | | <0.01 |  |
| **Mean Age,** (years)^a^,  N;(S.D) | 33.2,  (8.9) | 33.5,  (10.8) | 0.87 | 38.2,  (13.1) | 31.8,  (8.9) | | 0.05* | C |
| **Female**  N;(%) | 29,  (72.5) | 48;  (67.1) | 0.59 | 10,  (52.6) | 38,  (73.1) | | 0.10 |  |
| **UK**  N;(%) | 32;  (41.6) | 45;  (58.4) | 0.07 | 14;  (18.2) | 31;  (40.3) | | 0.10 |  |
| **Melancholic MDE^Χ^**  N;(%) | 0;  (0) | 18;  (25.4) | <0.01* | 18;  (94.7) | N/A | | <0.01* | A,B,C |
| **Psychotic MDE^Χ^**  N;(%) | 0;  (0) | 1;  (1.4) | <0.01* | 1;  (5.3) | N/A | | <0.01* | A,B,C |
| **Anxious-Distress MDE^Χ^**  N;(%) | 0;  (0) | 18;  (25.4) | <0.01* | N/A | 18;  (34.6) | | <0.01* | A,B,C |
| **Atypical MDE^Χ^**  N;(%) | 0;  (0) | 25;  (35.2) | <0.01* | N/A | 25;  (48.1) | | <0.01* | A,B,C |
| **Peripartum onset MDE^Χ^**  N;(%) | 0;  (0) | 1;  (1.4) | <0.01* | N/A | 1;  (2.0) | | <0.01* | A,B,C |
| **Unspecified MDE^Χ^**  N;(%) | 0;  (0) | 8;  (11.3) | <0.01* | N/A | 8;  (15.4) | | <0.01* | A,B,C |
| **BMI,**(weight/height^2^)^a^,  Mean(s.d) | 24.3,  (3.6) | 25.9,  (5.0) | 0.08 | 26.4,  (6.7) | 25.8,  (4.4) | | 0.19 |  |
| **Tobacco ^b^,**  N,(%) | 6,  (15.4) | 16,  (22.9) | 0.35 | 3,  (15.8) | 13,  (25.5) | | 0.46 |  |
| **Alcohol** (yes)  N; (%) | 31;  (79.5) | 53;  (74.7) | 0.56 | 14;  (73.7) | 39;  (75.0) | | 0.87 |  |
| **Contraceptive pill ^b^,**  N; (%) | 10,  (28.6) | 5,  (10.2) | 0.03* | 1;  (9.1) | 4,  (10.5) | | 0.10 |  |
| **Number Hair Washing**  *Per* week^a^, N;(%) | 4.5;  (1.7) | 4.3;  (2.0) | 0.50 | 3.7;  (1.6) | 4.5;  (2.2) | | 0.31 |  |
| **N. Episodes ^a^;**  Mean;(S.D) | 0;  (0) | 3.4;  (8.1) | <0.01* | 4.3;  (13.6) | 3.1;  (4.3) | | 0.02* | A,B |
| **N. Admissions ^a^;**  Mean;(S.D) | 0;  (0) | 0.2,  (0.5) | <0.01* | 0.1;  (0.3) | 0.2;  (0.6) | | 0.08 |  |
| **Duration of Illness ^a^**  in weeks; Mean;(S.D) | 0;  (0) | 86,  (119.75) | <0.01* | 84.3,  (109.2) | 92.4,  (142.9) | | <0.01* | A,B |
| **Age of illness started ^a^**  (years), Mean;(S.D) | 0;  (0) | 25.2,  (11.5) | <0.01* | 26.7;  (14.4) | 24.6;  (10.2) | | <0.01* | A,B |
| **Unipolar Disorder ^b^,**  N;(%) | 0;  (0) | 59,  (83.1) | <0.01* | 16;  (84.2) | 43;  (82.3) | | <0.01* | A,B |
| **Bipolar Disorder ^b^,**  N;(%) | 0;  (0) | 12,  (16.9) | <0.01* | 3;  (15.8) | 9;  (17.3) | | <0.01* | A,B |
| **HAMD-17^a^;**  Mean; (S.D) | 0.3;  (0.9) | 17.3,  (5.3) | <0.01* | 17.6;  (6.6) | 17.2;  (4.8) | | <0.01* | A,B |
| **Newcastle Scale**  Mean;(S.D) | 0;  (0) | 3.6;  (2.3) | <0.01* | 5.8,  (1.7) | 2.8,  (1.9) | | <0.01* | A,B,C |
| **YMRS ^a^;**  Mean;(S.D) | 0.1;  (0.3) | 1.2,  (1.7) | <0.01* | 0.9;  (1.2) | 1.3;  (1.8) | | <0.01* | A,B |
| **Early life trauma (CTQ)^b^**  N;(S.D) | 8;  (20.0) | 39,  (54.9) | <0.01* | 0.5;  (0.5) | 0.6;  (0.5) | | <0.01* | A,B |
| **Recent Life events (RLCQ)**  ≤3 months^a^, Mean;(S.D) | 100.7;  (162.1) | 319.7,  (294.5) | <0.01* | 314.2;  (159.9) | 321.8;  (334.3) | | <0.01* | A,B |
| **Severe RLCQ^b^**  ≤3 months, Mean;(S.D) | 6,  (15) | 47,  (66.2) | <0.01* | 15;  (79.0) | 32;  (62.0) | | <0.01* | A,B |
| **Mean ‘Hassles’^a^**  ≤1 month, Mean;(S.D) | 12.1;  (9.1) | 43.55;  (23.5) | <0.01* | 37.1;  (16.7) | 46.1;  (25.5) | | <0.01* | A,B |
| **N. Severe ‘Hassles’^b^**  ≤1 month, N;(S.D) | 1;  (2.5) | 35;  (49.3) | <0.01* | 7;  (36.8) | 28;  (54.0) | | <0.01* | A,B |
| ^M-MDE: Melancholic subtypes of MDE, NM-MDE: Non-Melancholic subtypes of MDE. Χ: MDE subtypes were obtained using DSM5 specifiers. A: Control ≠ M-MDE, B: Control≠ NM-MDE, C: NM-MDE≠ M-MDE *: P-value significant at 0.05 level a: t-test for continuous variable, b:^ *^Chi-^*^squared or Fisher tests for categorical variables. HAMD-17:17-item Hamilton Depression Rating Scale; CTQ: Childhood Trauma Questionnaire; RLCQ: Recent Life Change Questionnaire; YMRS: Young Mania Rating Scale; BMI: Body Mass Index^*^.^* ^HAMD-17: 17-item Hamilton Depression Rating Scale. ψ:^ *^Post hoc^* ^test used a Bonferroni correction. N/A: No Applicable.^ | | | | | | | | |

**Supplementary Table 2**: Cortisol level Comparison across controls, MDE and all MDE groups, using different specimens.

|  | **Groups** | |  | | | | |  |  |  |  |  |  |  |
| --- | --- | --- | --- | --- | --- | --- | --- | --- | --- | --- | --- | --- | --- | --- |
| **Cortisol**  **Measure** | **Controls** | **MDE** | *p-value_0_** | **MDE Groups** | | p-value_1_* | p-value_2_* | | *Post-hoc*  test^ψ^ | **MDE Sites** | | p-value_1_ | p-value_2_ | *Post-hoc*  test^ψ^ |
|  |  |  |  | **M-MDE** | **NM-MDE** |  |  |  |  | **UK-MDE** | **Chile-MDE** |  |  |  |
| **HCC**  (pg/mg),  mean,(s.d) | 8.3,  (3.9) | 8.3,  (4.6) | 0.96 | 8.8,  (4.1) | 8.1,  (4.8) | 0.82 | 0.70 | |  | 7.9;  (3.9) | 9.2;  (5.5) | 0.46 | 0.71 |  |
| **AUCg**  (nmol/l.h),  mean, (s.d) | 120.8,  (38.9) | 105.8,  (34.8) | 0.09 | 125.5,  (38.4) | 99.8,  (31.7) | 0.02* | 0.01* | | B | 117.4;  (32.3) | 80.5;  (26.0) | <0.01* | <0.01* | B,C |
| **CAR**  (nmol/l.h),  mean,(s.d) | 1.2,  (7.3) | 1.4,  (5.1) | 0.56 | 0.7,  (5.6) | 1.6,  (4.9) | 0.87 | 0.72 | |  | 2.5;  (5.0) | -1.1;  (4.3) | 0.13 | 0.07 |  |
| **DELTA**  (nmol/l.h),  mean,(s.d) | 2.3,  (6.7) | 2.9,  (7.1) | 0.73 | 1.4,  (9.0) | 3.3,  (6.6) | 0.67 | 0.87 | |  | 4.2;  (7.4) | -0.2;  (5.7) | 0.11 | 0.09 |  |
| ^*: P-values significant at 0.05 level.*P-value0 were obtained using Mann-Whitney U-test. P-values1 were obtained using ANOVA test P-values2 were obtained using Kruskal-Wallis test; HCC: Hair Cortisol Concentration; CAR: Cortisol Awakening Response, AUCg: Area Under the Curve with respect to the Ground; DELTA: the 30 minute delta cortisol secretion after awakening. A: Control ≠ M-MDE (UK-MDE), B: Control≠ NM-MDE (Chile-MDE), C: NM-MDE (UK-MDE)≠ M-MDE (Chile-MDE).^ | | | | | | | | | | | | | | |

**Supplementary Figure 1:** AUCg in M-MDE, NM-MDE and Control groups.


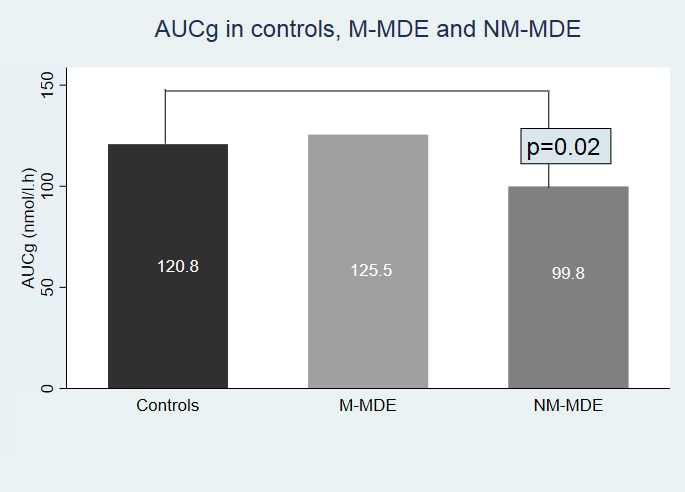


P-value was obtained using ANOVA test

**Supplementary Figure 2:** Associations between depression severity scores and long-(hair) and short-(saliva) cortisol output measures in MDE, M-MDE and NM-MDE groups.

**
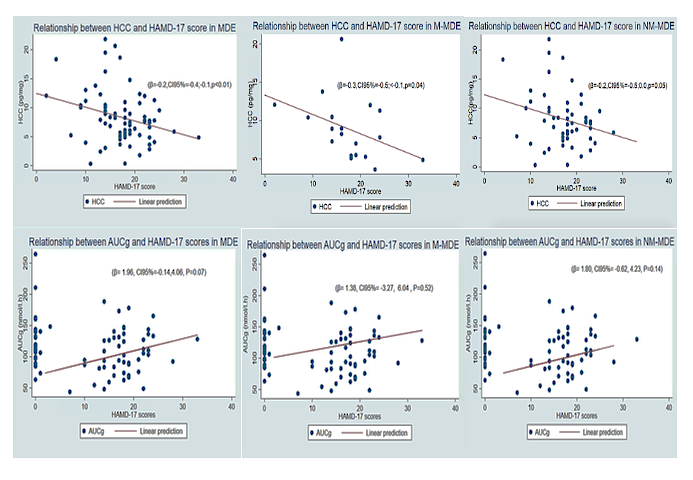
**

A Generalised Linear Model was used to study the association between HCC and HAMD-17 scores and Linear Regression Model was used to study the association between AUCg and HAMD-17 scores.

**Supplementary Figure 3:** Associations between depression severity scores and short-term cortisol reactivity measures (CAR and DELTA) in MDE, M-MDE and NM-MDE groups.

**
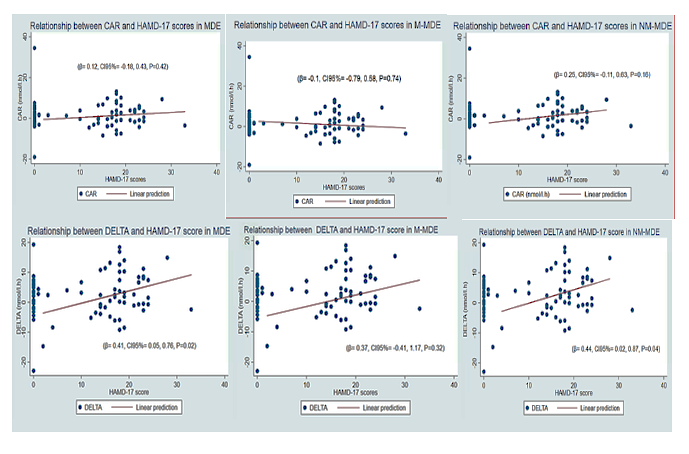
**

A Linear Regression Models were used to study the association between CAR & PEAK and HAMD-17 scores.

**Supplementary material**

**Hair collection procedures**

1. **Collection**

Hair samples were taken from the vertex at the back of the head and were cut with sterilised scissors as close to the scalp as possible. For this study, four locks of hair were required from different places from the vertex posterior, each to be the approximate thickness of a rubber band of 1 centimetre. At the laboratory, 3 cm of hair measured from the end to the scalp surface were cut from each lock, representing approximately 3 months of hair growth equivalent to 3-month retrospective assessment of cortisol production. The total weight of the four 3 cm segments from each lock is approximately equivalent to 60-80 mg of hair. Once collected, hair samples were stored at room temperature in the dark in a sealed container.

1. **Analysis**

Prior to analysis, the hair samples were washed in 1 ml of isopropanol to remove external contaminants, the isopropanol was removed from the vial and the hair allowed to dry in a clean air environment for 48 hours. Once fully dry five ceramic balls were added to each tube and the hair samples ground to a powder using am Fast Prep-24 (MP Biomedicals, LLC). To extract cortisol, 1.75 ml of methanol was added to each sample and the samples incubated for 20 hours whilst rotating the samples constantly.

The hair, methanol and ceramic balls were decanted into a polypropylene tube (Sarstedt AG & Co, Germany) that separated the ceramic balls from the rest of the mixture. The tube was centrifuged at 3000 RCF to separate the ground hair and methanol and 1.25ml of the clear methanol supernatant was decanted into a 2ml polypropylene cryovial. The methanol was then removed using a vacuum centrifuge (Scan Speed 40, Labgene) and the tubes frozen at -80°C until required for the cortisol ELISA. Cortisol levels were determined using a commercially available competitive ELISA (Salimetrics LLC, USA). Samples were thawed and reconstituted with 0.125ml of Salimetrics cortisol assay diluent and the samples were then assayed in accordance with the manufacturer’s protocol. The results were expressed as picograms of cortisol per milligram of hair. All hair samples were analysed at Salimetrics Laboratory, Cambridge, UK ([www.salimetrics.com](http://www.salimetrics.com)).

**Saliva specimen collection procedures**

1. **Collection**

Subjects were instructed not to smoke, brush their teeth, or have anything to eat or drink for at least an hour before the collection of the samples. Samples were collected (1) immediately after awakening, (2) 30 minutes after awakening, (3) 60 minutes after awakening, (4) at noon, (5) at 4pm, and (6) at 8 pm. Participants were instructed to avoid collections before 6 am and after 10 pm to minimise confounders. All participants filled out a questionnaire on socio-demographic details (gender, smoking habits, and health problems). Subjects were also instructed to specify whether they experienced any stressors and to provide any information which could be of relevance and/or interfere with the study. Moreover, they were asked to note the exact time for each saliva sample in a research log to assess self-reported compliance. Subjects were given instruction for storage and delivery the research team.

1. **Analysis**

Analyses of saliva cortisol concentrations were carried out in the Bethem Royal Hospital, London UK. On the arrival to the laboratory the salivettes were frozen at −20 ° Celsius. After thawing, they were centrifuged at 3500 rev/min for 10 min, which resulted in a clear supernatant of low viscosity. The saliva specimens were then frozen again in microtubes. Saliva cortisol concentrations were subsequently determined using the “Immulite” —DPC’s Immunoassay analyser (www.diagnostics.siemens.com). To plot a calibration graph, set of 22 cortisol standards in saline were used in each assay. Results were highly reproducible with mean slope of 0.197 and standard error of the mean (SEM) ± 0.004 and the method correlated well with a previously published Time-Resolved fluorescence immunoassay (TR-FIA) (57). It had analytical sensitivity of 0.2 nmol/l.h and inter/intra assay precision- total imprecision in percentage (% CV) was less than 10% (cortisol concentration range 5 to 25 nmol/l.h)-. All samples from the same subject were analysed in the same run.
